# Novel Hybrid Polymer Composites with Graphene and MXene Nano-Reinforcements: Computational Analysis

**DOI:** 10.3390/polym13071013

**Published:** 2021-03-25

**Authors:** Sigitas Kilikevičius, Saulė Kvietkaitė, Leon Mishnaevsky, Mária Omastová, Andrey Aniskevich, Daiva Zeleniakienė

**Affiliations:** 1 Department of Mechanical Engineering, Kaunas University of Technology, Studentų st. 56, 51424 Kaunas, Lithuania; saule.kvietkaite@ktu.lt (S.K.); daiva.zeleniakiene@ktu.lt (D.Z.); 2 Department of Wind Energy, Technical University of Denmark, 2000 Roskilde, Denmark; lemi@dtu.dk; 3 Polymer Institute, Slovak Academy of Sciences, Dúbravská cesta 9, 845 41 Bratislava 45, Slovakia; maria.omastova@savba.sk; 4 Institute for Mechanics of Materials, University of Latvia, Jelgavas st. 3, LV-1004 Riga, Latvia; andrey.aniskevich@pmi.lv

**Keywords:** hybrid composites, MXene, graphene, modelling, damage

## Abstract

This paper presents a computational analysis on the mechanical and damage behavior of novel hybrid polymer composites with graphene and MXene nano-reinforcements targeted for flexible electronics and advanced high-strength structural applications with additional functions, such as real-time monitoring of structural integrity. Geometrical models of three-dimensional representative volume elements of various configurations were generated, and a computational model based on the micromechanical finite element method was developed and solved using an explicit dynamic solver. The influence of the geometrical orientation, aspect ratio, and volume fractions of the inclusions, as well as the interface properties between the nano-reinforcements and the matrix on the mechanical behavior, was determined. The results of the presented research give initial insights about the mechanical and damage behavior of the proposed composites and provide insight for future design iterations of similar multifunctional materials.

## 1. Introduction

After the discovery of graphene, focus shifted to two-dimensional (2D) nanomaterials. These nanofillers drew attention due to their flexible properties, allowing us to create multi-functional composite materials that can be used for a range of applications such as aerospace, energy storage, and electromagnetic interference shielding [1,2,3]. Two-dimensional nanomaterials such as graphene, due to their high aspect ratio (AR), offer increased fatigue resistance and fracture toughness through deflection of crack propagation and bridging mechanics [4,5,6]. However, such fillers are hydrophobic and do not form bonds with polymers. Such a pursuit led to the discovery of a new class of 2D materials in 2011. MXenes are ternary layered compounds, produced by selectively etching A-group layers from the MAX phases. They exhibit unique properties such as hydrophilic nature combined with high electrical and thermal conductivity, capability to intercalate ions, high electrical capacity, excellent electrochemical activity, and great mechanical properties [7,8,9,10,11,12,13,14,15]. These properties make them of great interest for many applications, and they can be utilized in developments of composite materials with polymer matrixes for flexible electronics and advanced high-strength structural applications with additional functions such as real-time monitoring of structural integrity. There are more than 30 different types reported, and hundreds computationally studied in silico [7,16]. Titanium carbide Ti_3_C_2_T_z_ is the most widely researched MXene exhibiting hydrophilic properties, which makes it dispersible in a range of polar solvents [17,18]. Ti_3_C_2_T_z_ of high aspect ratios obtained by using several different methods showed increased conductivity [19,20,21], as well as improved thermal [22] and mechanical properties [23] of polymer composites. Moreover, these materials showed great promise for developing materials with long-term thermo-oxidative resistance [24]. Furthermore, recent studies demonstrate high adhesion between MXene and epoxy resin [25].

The mechanical properties of the hierarchical composite greatly depend on 2D nanofiller geometry (shape, length, and thickness). Depending on the synthesis method of the MXene, varying thickness and number of layers can be obtained [19,26]. The average thickness of a single Ti_3_C_2_T_z_ flake is 0.8 nm, while a thickness of 1 nm is characteristic for the flake with surface functional groups, and the mean lateral size is usually around 500 nm [26,27]. As multi-layered MXenes are more common, their thickness can range from 5 to 30 nm with AR 17-100 [28,29,30,31].

Recently, the electrical properties of polymer composites have gained great interest. The conductivity properties of hybrid composites with carbon nanotubes and carbon black can by utilized for energy storage [32]. Carbon nanotubes and carbon fiber were studied to implement electro-activated polymeric shape-memory nanocomposites [33,34,35]. Moreover, nano-reinforcements in polymer-based composites can be utilized for strain sensing [36,37,38,39]. All this shows a huge potential to expand the scope of applications of polymer composites. With this in mind, MXenes electrical properties can be utilized in polymer composites with additional functions such as real-time monitoring of structural integrity.

The finite element-based approach was proven to be a very valuable tool for studying the mechanical and damage behavior of various composite materials. In the field of finite element-based analysis on nanocomposites, the majority of published works are focused on graphene for improving the mechanical characteristics of nanocomposites [40,41,42,43,44,45,46,47,48,49,50,51,52]. Recently, the finite element-based methods gained attention in studying the damage behavior of various composites. The finite element method along with the molecular dynamics model were used for modelling the fracture and strength of single layer graphene platelets reinforced reacted epoxy [53]. The mechanical properties of epoxy and interfaces between graphene and epoxy were obtained by modelling crosslinking reactions with the molecular dynamics model. These properties were used in the finite element model to investigate the effects of graphene morphology on the composites. Computational micromechanics in conjunction with the augmented finite element method were applied for an investigation on damage mechanisms in thin-ply composite laminates [54]. The finite element method was applied for analyzing the progressive failure of open-hole composite laminates for aeronautical applications and demonstrated a good agreement with the experiments [55]. A numerical model of tensile response and damage evolution in flax/epoxy and carbon/epoxy composites was developed within a thermodynamics framework, and the predictions made by this model corelated well in terms of mechanical response, stiffness degradation, and inelasticity [56]. The damage and fracture mechanisms of graphene/epoxy composites were researched in another work [57]. The influence of the shape, aspect ratio, orientation, clustering, and volume fraction of graphene reinforcements was demonstrated by computational experiments based on the finite element method. The finite element-based micromechanics study of epoxy composite reinforced with pristine graphene and reduced graphene oxide nanoplatelets [58] demonstrated that the rivalry between the brittle matrix cracking and interface debonding damage mechanisms is influenced by the orientation of nanoplatelets, volume fractions, nanoplatelets/matrix modulus-mismatch, and interface strength. The same approach was used for an analysis on the damage behavior of a nacre-inspired graphene oxide/polyvinylidene fluoride nanocomposite [59]. Crack deflection and excessive plastic deformation was observed when fractions of graphene were lower, while brittle fracture was observed when fractions of graphene were higher due to the coalescence of cracks. Finite element-based micromechanical models were applied to study the influence of functionally graded voids and graphene nanoplatelets on the damage behavior of polyurethane foam core [60]. The study revealed that the air pores significantly increased the ductility of brittle thermoplastic polyurethane resin when the pores were distributed non-linearly in a functionally graded circular shape, and graphene nano-reinforcements compensated the decrease in the Young’s modulus occurring due to linearly distributed air-voids.

The tensile response and the damage mechanism of MXene/epoxy composites MXene/polyvinyl alcohol composites were investigated [61] by developing a micromechanical finite element model, which was calibrated taking into account experimental results. The predictions based on this model demonstrated that MXene shows great promise for polymer matrix-based composites by significantly improving their mechanical properties, as well as high-strength multifunctional MXene-polymer film materials with high mechanical properties, which can be applied for real time monitoring of structural integrity by utilizing the electrical conductivity of MXene. Bioinspired MXene/polymer nanocomposites with nacre mimetic brick and mortar structures were modelled using classical analytical methods and numerical methods based on the finite element approach [62]. It was demonstrated that such structures result in an interlocking mechanism between MXene inclusions, leading to a significant increase in stiffness and strength. Orthotropic elastic properties of epoxy composites with MXene and graphene 2D nano-reinforcements were studied [63] by applying numerical methods. Recent works demonstrated that the mechanical and damage behavior of composites can be significantly improved by proper selection of reinforcements.

The aim of this research is to study the mechanical and damage behavior of hybrid polymer composites with MXene and graphene nano-reinforcements by developing a computational model based on the micromechanical finite element method, which would allow initial insights on the mechanical and damage behavior of such hybrid composites to be presented and estimations to be made about the influence of the geometrical orientation, aspect ratio and volume fractions of the inclusions as well as the interface properties between the nano-reinforcements and the matrix.

## 2. Materials and Methods

The investigated composite materials are composed of an epoxy matrix and 2D nanosheets of graphene and MXenes. The mechanical properties of the materials are presented in Table 1. The graphene-matrix interphase properties used in the presented computational analysis were based on the previous research [25,57], which studied these properties using an inverse modelling approach.

To investigate the mechanical behavior of such hybrid polymer composites reinforced with graphene and MXene nanosheets, a computational model was developed on the basis of the micromechanical finite element method. Geometrical models of three-dimensional representative volume elements (RVEs) with various volume fractions (denoted as *f_G_* and *f_MX_*, respectively) of graphene and MXene inclusions, various aspect ratios (*ρ_G_* and *ρ_MX_*), and different alignment configurations were created using Digimat-FE (Extreme Engineering, MSC.Software GmbH, Munich, Germany). In practice, the alignment of nano-reinforcements can be achieved through electrical methods [68,69]. A volume fraction for graphene inclusions was set to 0.1%. The MXene volume fractions were set in a range from 0.8% to 1.6% for the RVEs with randomly placed inclusions and in a range from 0.8% to 6.4% for the RVEs with aligned inclusions. An aspect ratio value of 500 was used for the graphene inclusions, while aspect ratio values of 200 and 400 were used for the MXene inclusions.

**Figure 1:**
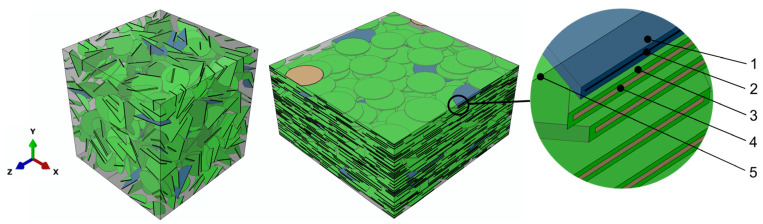
Typical representative volume elements (RVEs) with random placed inclusions (*ρ_G_* = 500, *ρ_MX_* = 125, *f_G_* = 0.1%, and *f_MX_* = 1.6%) and aligned inclusions (*ρ_G_* = 500, *ρ_MX_* = 125, *f_G_* = 0.1%, and *f_MX_* = 6.4%): (**1**), graphene/matrix interface; (**2**), graphene; (**3**), MXene/matrix interface; (**4**), MXene; (**5**), matrix.

The RVEs with randomly placed inclusions were built as cubes with a size of 595 nm, while the RVEs with aligned inclusions were built as rectangular cuboids with dimensions of 595 × 290 × 595 nm, aligning the inclusions in the x–z plane. The graphene and MXene inclusions were generated as discs with a thickness of 0.335 nm and 1 nm [1,70,71,72], respectively. As inclusions/polymer matrix interfaces make a significant influence on the mechanical behavior of composite materials reinforced with nanosheets, the approach of effective interface models was adopted where the thin layer, surrounding the inclusions, is generated with specific properties. Based on the experimental observations presented in the article [73], the thickness of the effective interface layers was set to 1 nm. Typical RVEs used in this research are presented in Figure 1.

The created RVEs were imported to the commercial finite element software Abaqus FEA (Dassault Systemes, Vélizy-Villacoublay, France), which was used to develop computational model and carry out the simulation tasks. The periodic boundary conditions [74] were opted in Digimat-FE (Extreme Engineering, MSC.Software GmbH, Munich, Germany) and were imported along with the geometrical models to Abaqus FEA (Dassault Systemes, Vélizy-Villacoublay, France). The RVEs were subjected to uniaxial tensile loading along the x-axis direction [61,63] The RVEs were meshed using the three-dimensional 4-node linear tetrahedron element (C3D4) type. A minimum size of 8 nm was applied for the mesh, resulting in a total number of 1–2 million, depending on the RVE’s configuration. The experimental stress–strain curve of epoxy [67] was inserted in the program and the multilinear hardening plasticity model was considered to define the response to the mechanical loading. To investigate the influence of the MXene/epoxy interface properties on the strength of the proposed composite, several values of the Young modulus *E_MX_* and strength of the interface was used (multiplying by 0.5, 0.75 and 1.5 to those of the matrix *E_m_*). These values were in the range determined in the previous research [61]. The maximum principal stress criterion was applied for the simulation of matrix and interfaces cracking using the values provided in Table 1 as it was demonstrated that these are reasonable value for the maximum principal strength [75,76]. The MXene and graphene inclusions were not damaged during the simulation as the obtained maximum principal stress did not exceed the strength limit of these materials. The developed computational model was solved using Abaqus explicit and converged, delivering reliable and stable results.

## 3. Results and Discussion

**Figure 2:**
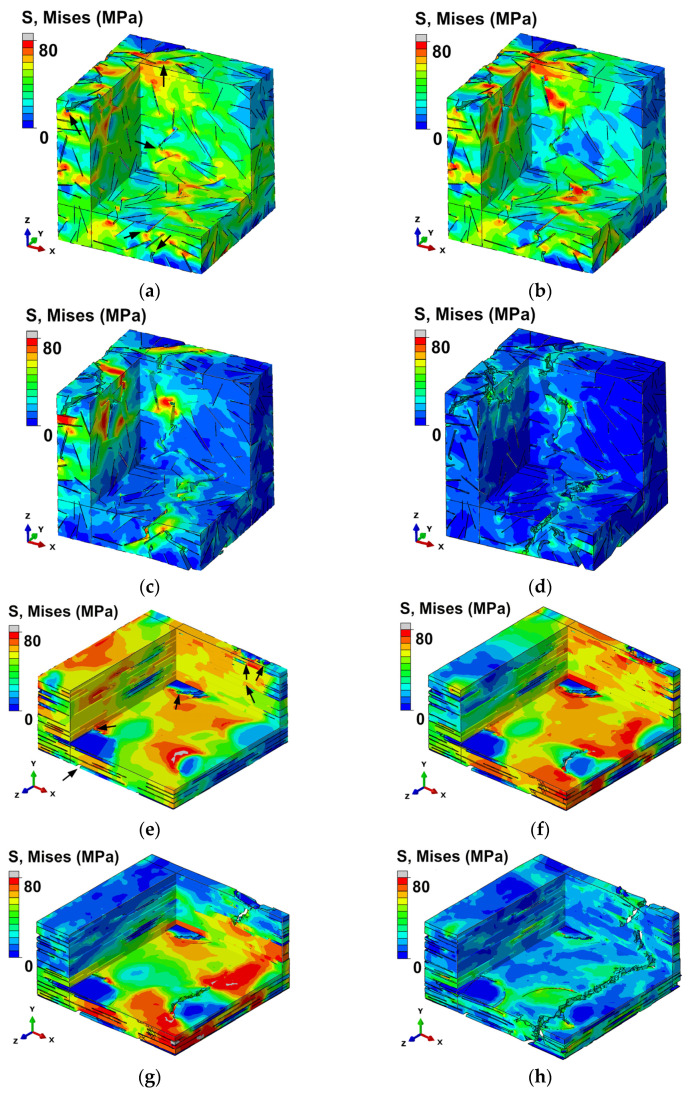
Stress distribution inside RVEs demonstrating crack formation and propagation (cut views): (**a**) RVE with randomly placed inclusions at a strain of 0.026, *ρ_G_* = 500, *ρ_MX_* = 125, *f_G_* = 0.1%, *f_MX_* = 1.6%, and *E_MX_* = 0.5*E_m_* (localized cracking is indicated by the black arrows); (**b**) at a strain of 0.029; (**c**) at a strain of 0.031; (**d**) completely fractured at a strain of 0.038; (**e**) the RVE with aligned inclusions at a strain of 0.032, *ρ_G_* = 500, *ρ_MX_* = 250, *f_G_* = 0.1%, *f_MX_* = 3.2%, and *E_MX_* = 0.5*E_m_* (localized cracking is indicated by the black arrows) at a strain of 0.036; (**f**) at a strain of 0.039; (**g**) at a strain of 0.041; (**h**) completely fractured at a strain of 0.045.

**Figure 3:**
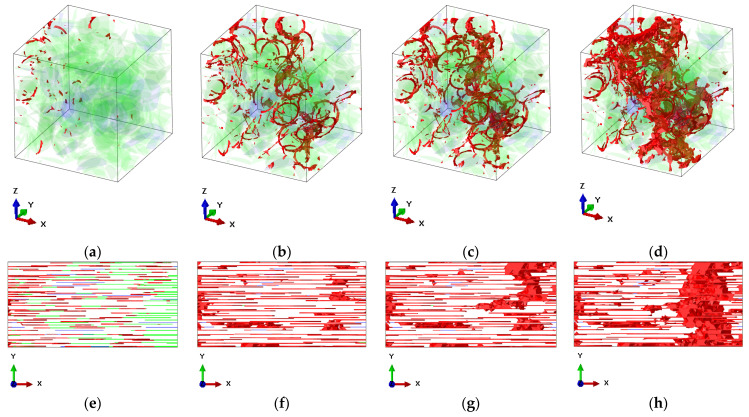
Damage evolution in the RVEs (damage is highlighted in red): (**a**–**d**) the RVE with randomly placed inclusions (the interface layers are hidden for better crack visualization, the MXenes are shown in transparent green, while the graphene inclusions are shown in transparent blue), *ρ_G_* = 500, *ρ_MX_* = 125, *f_G_* = 0.1%, *f_MX_* = 1.6%, and *E_MX_* = 0.5*E_m_*: (**a**) at a strain of 0.016; (**b**) at a strain of 0.026; (**c**) at a strain of 0.029; (**d**) at a strain of 0.038; (**e**–**h**) the RVE with aligned inclusions (the MXenes are shown in green, while the graphene inclusions are shown in blue), *ρ_G_* = 500, *ρ_MX_* = 250, *f_G_* = 0.1% *f_MX_* = 3.2%, and *E_MX_* = 0.5*E_m_*: (**a**) at a strain of 0.011; (**b**) at a strain of 0.036; (**a**) at a strain of 0.039; (**a**) at a strain of 0.045.

Stress distributions in RVEs subjected to tension along the x-axis direction are shown in Figure 2 and damage evolution is shown in Figure 3.

At the beginning, the MXene/epoxy interfaces start to fail (Figure 3e). High-stress concentrations at the edges of the nano-reinforcements were observed (Figure 2a,e) resulting in the formation of localized cracking at the edges of the nano-reinforcements in both RVEs (Figure 3a,e). In the RVE with randomly placed inclusions, matrix damage was observed at a strain value of 0.016 (Figure 3a). As the strain increases, the main crack starts to form (Figure 2b and Figure 3b) and propagate (Figure 2c and Figure 3c). A complete fracture of the RVE with randomly placed inclusions was observed at a strain of 0.038 (Figure 2d). In contrast, in the case of the RVE with aligned inclusions, it was observed at a strain of 0.045 (Figure 2h). After a complete fracture (Figure 3d,h), the stress dropped, and crack pinning and deflection of the epoxy matrix were observed in the fractured RVEs (Figure 2d,h).

**Figure 4:**
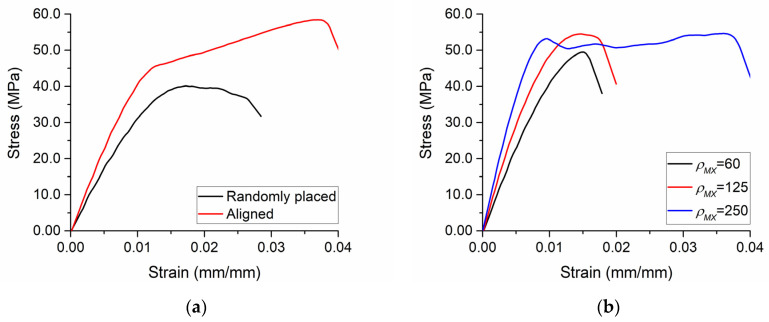
The influence of: (**a**) geometrical orientation of the inclusions at *ρ_G_* = 500, *ρ_MX_* = 125, *f_G_* = 0.1%, *f_MX_* = 1.6%, and *E_MX_* = 0.5*E_m_*; (**b**) the aspect ratio of aligned MXene inclusions at *ρ_G_* = 500, *f_G_* = 0.1%, *f_MX_* = 3.2%, and *E_MX_* = 0.5*E_m_*.

The influence of geometrical orientation of the inclusions is shown in Figure 4a. Both the effective Young’s modulus and the tensile strength were higher in the case of aligned MXene and graphene inclusions. In the composites containing *ρ_G_* = 500, *ρ_MX_* = 125, *f_G_* = 0.1%, *f_MX_* = 1.6%, and *E_MX_* = 0.5*E_m_*, the effective Young’s modulus was 3.4 GPa, and the tensile strength was 40.1 MPa with randomly placed inclusions, while the effective Young’s modulus along the alignment (x-axis) direction was 4.6 GPa and the tensile strength was 58.4 MPa with aligned inclusions. The influence of the aspect ratio of aligned inclusions is shown in Figure 4b, at *ρ_G_* = 500, *f_G_* = 0.1%, *f_MX_* = 3.2%, and *E_MX_* = 0.5*E_m_*. At MXene aspect ratio values of 60, 125, and 250, the effective Young’s moduli were 4.7 GPa, 6.17 GPa, and 8.1 GPa, respectively. Additionally, higher values of elongation at break were observed at higher values of aspect ratio.

**Figure 5:**
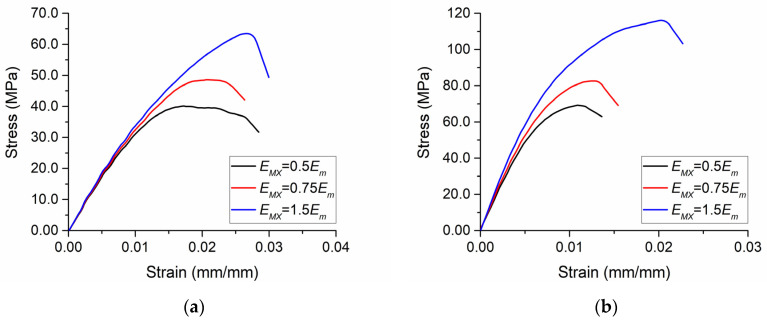
The influence of the MXene/epoxy interface: (**a**) randomly placed MXenes, *ρ_G_* = 500, *ρ_MX_* = 125, *f_G_* = 0.1%, and *f_MX_* = 1.6%; (**b**) aligned MXenes, *ρ_G_* = 500, *ρ_MX_* = 125, *f_G_* = 0.1%, and *f_MX_* = 6.4%.

The MXene/epoxy interface did not have a significant influence on the effective Young’s modulus of the composite; however, higher properties of the interface result in significantly higher values of the tensile strength (Figure 5). In the RVEs with randomly placed MXenes having *ρ_G_* = 500, *ρ_MX_* = 125, *f_G_* = 0.1%, and *f_MX_* = 1.6%, the tensile strength was 40.1 MPa, 48.6 MPa, and 63.5 MPa, at *E_MX_* = 0.5*E_m_*, *E_MX_* = 0.75*E_m_*, and *E_MX_* = 1.5*E_m_*, respectively (Figure 5a). In RVEs with *ρ_G_* = 500, *ρ_MX_* = 125, *f_G_* = 0.1%, and *f_MX_* = 6.4%, where MXenes aligned, the tensile strength was 69.2 MPa, 82.6 MPa, and 116.1 MPa, at *E_MX_* = 0.5*E_m_*, *E_MX_* = 0.75*E_m_*, and *E_MX_* = 1.5*E_m_*, respectively (Figure 5b). Moreover, elongation at break was almost two times higher when *E_MX_* = 1.5*E_m_* compared to the case when *E_MX_* = 0.5*E_m_*.

These results confirm the previous findings [63] that the elastic properties of this composite are not highly influenced by the MXene/epoxy interface properties. However, this research shows that the interface has an influence on the fracture response.

**Figure 6:**
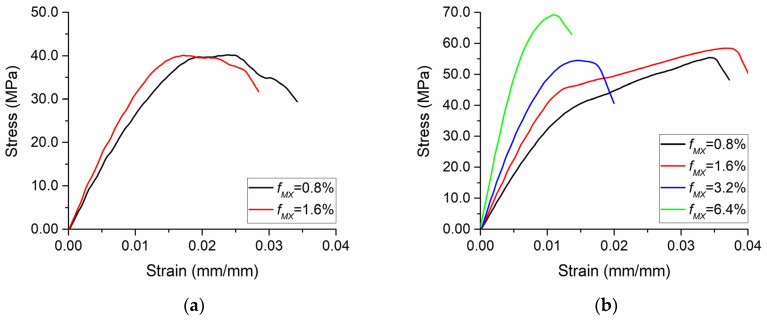
The influence of volume fractions of MXenes: (**a**) randomly placed MXenes, *ρ_G_ =* 500, *ρ_MX_* = 125, *f_G_* = 0.1%, and *E_MX_* = 0.5*E_m_*; (**b**) aligned MXenes, *ρ_G_* = 500, *ρ_MX_* = 125, *f_G_* = 0.1%, and *E_MX_* = 0.5*E_m_*.

The influence of the MXene volume fraction is shown in Figure 6. An increase in the volume fracture resulted in a significant increase in the effective Young’s modulus of the composite. With randomly placed inclusions as *ρ_G_* = 500, *ρ_MX_* = 125, *f_G_* = 0.1%, and *E_MX_* = 0.5*E_m_*, the obtained effective Young’s moduli were 2.8 GPa and 3.4 GPa at *f_MX_* = 0.8% and *f_MX_* = 1.6%, respectively. For the composites with aligned MXenes, containing *ρ_G_* = 500, *ρ_MX_* = 125, *f_G_* = 0.1%, and *E_MX_* = 0.5*E_m_*, effective Young’s modulus values of 3.5 GPa, 4.6 GPa, 6.1 GPa, and 10.1 GPa were obtained at *f_MX_* = 0.8%, *f_MX_* = 1.6%, *f_MX_* = 3.2%, and *f_MX_* = 6.4%, respectively.

Summarizing the results in Figure 5 and Figure 6, it should be noted that a stronger interface is able to improve both the maximum stress and the maximum strain, while the volume fraction of MXene is able to improve the maximum stress but the maximum strain is then decreased as the composite becomes more brittle. When developing compositions of new hybrid polymer composites with graphene and MXene nano-reinforcements, it is important to take these insights into account and control the manufacturing procedures in order to ensure a proper interface as well as optimize the MXene volume fraction in such cases where the degradation of structures occurs at small deformations.

Besides load carrying capabilities, the proposed composite can utilize the electrical properties of MXene. For example, when a structure made of such composite loses its structural integrity, the electrical conductivity is also deteriorated. This can be easily measured in real-time and give a reference about the structural integrity state.

## 4. Conclusions

Novel hybrid polymer composites with graphene and MXene nano-reinforcements were proposed, and a computational model based on the micromechanical finite element method was developed for studying the mechanical and damage behavior of hybrid polymer composites with MXene and graphene nano-reinforcements.

The influence of the geometrical orientation, aspect ratio and volume fractions of the inclusions, as well as the interface properties between the nano-reinforcements and the matrix, on the mechanical behavior was studied. The modelling demonstrated that both the effective Young’s modulus and the tensile strength were higher in the composites with aligned MXene and graphene inclusions in comparison to the composites with randomly placed inclusions. An increase in the volume fracture of the nano-reinforcements results in a significant increase in the effective Young’s modulus of the analyzed composites. The effective Young’s modulus of the composite with aligned nano-reinforcements containing 0.1% and 6.4% volume fractions of graphene and MXene, respectively, was 3.65 times higher compared to the Young’s modulus of the matrix. Moreover, the tensile strength increased as the volume fraction of MXene increased in the composites with aligned nano-reinforcements. However, in the composites with randomly placed nano-reinforcements, an increase in the volume fraction resulted in only an increase in the effective Young’s modulus. The MXene/epoxy interface did not have a significant influence on the effective Young’s modulus of the composite; however, higher properties of the interface resulted in significantly higher values of the tensile strength. Further, higher mechanical properties were observed at higher aspect ratios of nano-reinforcements. Crack pinning and deflection of the epoxy matrix were observed in the fractured RVEs.

Graphene nano-reinforcements may be used for additional strengthening of multifunctional composites with MXenes and further expand the scope of application of such materials by utilizing the great mechanical properties and electrical conductivity of MXene. The proposed novel hybrid polymer composites with graphene and MXene nano-reinforcements can be applied for flexible electronics and advanced high-strength structural applications with additional functions as real-time monitoring of structural integrity.

The results of the computational analysis revealed that MXene and graphene nano-reinforcements demonstrate considerable promise in the development of novel multifunctional composites, exhibiting excellent mechanical properties.

## Figures and Tables

**Table 1:** The mechanical properties of the materials.

	Materials			
Material Property	MXene (Ti_3_C_2_) [64,65]	Graphene [66]	Graphene/Epoxy Effective Interface [25,57]	Epoxy [67]
Young’s modulus (GPa)	330	1000	3.74	2.74
Poisson’s ratio	0.23	0.165	0.35	0.35
Strength (MPa)	22,000	130,000	120	80.3
Elongation at break (%)	5.5	20		5.3
